# Book Reviews

**Published:** 1823-01

**Authors:** 


### CRITICAL ANALYSIS
OF
### ENGLISH AND FOREIGN LITERATURE
RELATIVE TO THE VARIOUS BRANCHES OF
### Medical Science.

Quæ laudanda forent, et quæ culpanda, vicissim

Illa, prius, cretâ; mox hæc, carbone, *notamus*.—PERSIUS.

### DIVISION I.

### ENGLISH.

ART. I.—*Practical Observations on Distortions of the Spine, Chest, and Limbs; together with Remarks on Paralytic and other Diseases connected with impaired or defective Motion.* By WILLIAM WARD, F.L.S. Member of the Royal College of Surgeons, of the Medico-Chirurgical Society, and Fellow of the Medical Society of London.—8vo. pp. 168. T. and G. Underwood, London, 1822.

WE have perused this volume with considerable pleasure, and, independently of its intrinsic merit, we can also recommend it to our readers as an excellent specimen of a plain, perspicuous, unaffected style of composition, which in the present day is rather a rarity; many modern authors (young ones especially,) appearing rather to study how to render their phrases involved, encumbering their language with false ornament, and overwhelming us with needless authority and quotation. As a proof of the opinion we entertain of Mr. WARD's book, it is our intention to give very few remarks of our own, but to present a concise, and we trust faithful, analysis of the body of the work, —a great part of which (perhaps too much,) is occupied in the detail of cases.

In a short preface, our author explains to us the object he has in view. He urges, that the exemption from disease, enjoyed by uncivilized nations, " is in no small degree" attributable to bodily labour, and "that its importance in the preservation of health has been a matter of observation from the earliest periods. Notwithstanding this assent to its general utility, the application of this power in the recovery of natural, or the removal of the disordered function of particular parts of the body, seems to have been little attended to. To supply, in some degree, this deficiency, by combining theoretical views with practical results, is the object of the present attempt. He next states that the treatment he recommends was at first suggested to him by Mr. PUGH's work on Muscular Motion; and he gives it as his opinion, in which we entirely concur with him, that the application of this principle, by carrying a weight on the head, is better adapted to the slighter cases of curvature of the spine, whether anterior or lateral, than to those of great extent or long duration ; and may be resorted to as an auxiliary when the spine has become nearly restored to its original shape.

The first chapter, which treats on the influence of muscular exercise on the body, we may be allowed to pass over, until we come to the inferences our author has deduced, and which we give, not on account of their novelty, but because his mode of treatment is, in a great degree, founded upon these principles; and, therefore, their insertion is necessary to the understanding of the author's practice.

"From what has been stated, I think we may draw the following inferences:

"That the comparative power of muscular parts depends,

"1. On the state of the functions of respiration and circulation, and that increased strength is a consequence of increased vascularity and circulation of blood in the part; and, *vice versa*, a want of tone and power, of a deficient supply of it.

"2. On the degree of exercise or frequency with which they are called into action.

"3. On the mental energy or power of volition exerted on them.

"4. That the most effectual means of increasing muscular strength is by the frequent exercise of the power itself, and, consequently, the preservation of the healthy actions of those functions by which it is influenced.

"5. That the muscular parts have a constant tendency to contract, by which they adapt themselves to the state of the limb or parts to which they are attached.

"The proper application of these principles appear to me of essential importance in the treatment of those disordered states of the limbs which are accompanied with muscular weakness, more especially as it is often associated with diseases which not only affect the comfort, but the life, of the individual."

Chapter 2d, *Curved Spine.*—Leaving the definition of this disease, we find Mr. Ward describing, as usual, two kinds of curvatures of the spine,—the lateral and anterior; the former occurring more frequently during the growth of young persons; the latter at a more advanced period of life, as one of the sequelæ of chronic rheumatism, or of any long protracted disorder occasioning muscular weakness. The appearances on dissection are the following:—The intervertebral substance is thinner than natural, especially on the concave side of the curve; the ligaments are not so strong as in a healthy subject; the transversalis muscles inserted into the spinous processes are elongated, and much finer and smaller on the convex than the concave side of the curve, where they are shorter and fuller. We believe with our author, that this disease, the lateral curvature especially, is increasing in frequency; and he attributes this, in part, to that fatal refinement in female education, which substitutes showy accomplishments for sound health, and he justly reprobrates the funereal pace and solemn march of a modern boarding-school, taking what is called exercise. He strongly urges mothers to suckle their infants, believing the complaint frequently to originate in the neglect of this important duty; and his remarks upon the over-feeding of infants after they are weaned, are no less important, and, we may add, not a whit less applicable to "children of a larger growth:" at least, his question, whether diseases arising from privation of food are not less frequent than those occasioned by the contrary extreme? will, we believe, be pretty generally answered in the affirmative.

It has been supposed by many, says Mr. Ward, that certain habits, such as standing on one leg, sitting awry, &c. have a share in producing distortion; but he is disposed to believe these habits to be rather signs indicating that an alteration has already taken place in the relative position of the trunk, and that these postures are only, in fact, efforts to preserve the equilibrium of the body.

We do not quite agree with our author, that the "opinions generally entertained upon the subject of distorted spine, appear to have been that it has always had its origin in caries of the vertebræ, or in a morbid state of the bone tending to it." Indeed, we believe rather the contrary to be the fact, and that it is pretty universally allowed that the lateral and anterior curvatures of the spine arise solely from a softness of the bone, generally by no means implying either caries or a disposition to it. His description of the mode in which the disease comes on, the sense of lassitude, the disinclination to move, and the impaired digestion, which precede any evident or apparent alteration of shape, are accurately described; and he accounts rationally, and we think satisfactorily, for the curvature more commonly taking place towards the right than the left side, although there is nothing very novel in this explanation.

"That to muscular debility (says Mr. Ward,) we may ascribe the first occurrence of the disease, is confirmed by the method of cure, which is founded on the principle of giving increased action to those muscles of the spine which have been weakened in the incipient state of the disease." It is too often proved that parents, who are quick-sighted enough to detect the smallest deviation in shape, undertake, with the aid of instruments, or stays, to correct the evil. Nothing can be more pernicious: it is beginning at the wrong end; and, if ever such means are justifiable, it can only be when the disposition to the disease is broken, and as a means of perfecting the cure when the erect posture is permitted. Contrary to what takes place in the angular curvature, which is the direct consequence of ulceration in the intervertebral substance, or the bone itself, in the lateral and anterior curvatures of the spine, the complaint comes on very gradually: there is no acute pain; sometimes a sense of uneasiness, but which does not, in general, seem to be referrible to the bone, in the angular curvature. It is true, as Mr. Ward says, that there is a sudden projection of the part; but there is great truth, also, in what Mr. COPELAND has urged upon this point, that the existence of the disease in the spinal column may be detected often, by a careful examination, long prior to any apparent deformity; and his method of sponging in the course of the vertebral column with hot water, will very often point out at once where the malady exists.

"The curvature of the spine anteriorly, as a sequel of chronic rheumatism, or any other long-protracted debilitating disease, is not an unfrequent complaint. It appears to be induced by the patient inclining forward when sitting or laying, with a view to procure relief from pain:" and these instances are especially adapted for the practice advocated by Mr. Ward. The principle of cure is to restore the balance of power between the muscles contracted and those in a state of extension; and these means our author divides into passive and active. Under the head of *active* are friction, shampooing, percussion, galvanism, electricity, confinement to particular postures, &c.; the *passive* consists of the excitement of muscular action by exercise. We agree with Mr. Ward in his condemnation of the inclined plane, in both the lateral and anterior curvatures; and we think the horizontal posture, upon a good hard mattress, much more agreeable to the patient, allowing of many partial changes of posture, which relieve the weariness of particular muscles, without at all interfering with the benefits to be derived from the recumbent position.

We now come to the application of the active measures which our author recommends, and which we shall therefore give in his own words:

"This intention will be best attained by putting in force the measures already hinted at under the head of active treatment, which consists in compelling the muscles to exert themselves with energy to restore the spine to its natural situation. One of the methods that I employ for this purpose, and the detail of which will place the subject in the clearest point of view, is the following:—a weight appended to a cord is passed over a pully, and the other extremity, having a strap attached to it, is fastened round the patient's head; the pelvis being fixed, the patient is directed to raise the weight by drawing the head and trunk backwards, and to repeat this effort until fatigue is produced. The frequency of repetition of this exercise of the muscles, and the weight of the body to be raised, must, of course, depend upon the patient's strength. After each effort, it is adviseable to take rest, by laying down on a couch or sofa, in order that the muscles may not be placed on the stretch, and thus prevented from recovering themselves. This mode of exercising the muscles is equally applicable to the anterior curvature of the spine, as to those which take place laterally.

"A combination of these means of muscular excitement will be attended with more advantage than when pursued separately. I have witnessed several cases in which friction alone has been unsuccessfully employed for a considerable length of time, and others where the inclined plane also has been depended upon solely, without the other measures being prosecuted at the same time, in which a combined plan of percussion and strong muscular exertion, assisted by a recumbent posture, has afterwards been attended with complete success."

We cannot dismiss this part of our author's work without some allusion to another labourer in the same field; and in mentioning Mr. BAMPFIELD's paper in a late Number of this Journal, we would remark, that it is but the commencement of a series which promises to be of great interest.

Seven cases, in illustration of Mr. Ward's practice, are next detailed, some of them at considerable length, but which we have not space to insert. The first, however, shows, in a very striking manner, the benefit that may be derived by steady perseverance and assiduous friction, with other means, under circumstances apparently the most hopeless.

The third chapter is entitled "*Of Deformity of the Chest,*" which, although generally the consequence of some deviation from the natural shape of the vertebral column, sometimes exists (says our author,) independently of any such affection. It is well known that this deformity of the chest is familiarly called being *chicken breasted;* and it may take place either by a projection of the sternum, in consequence of the flattening of the ribs on each side; or there may be a falling-in, instead of a projection, of the sternum; or, finally, one side of the breast may be flattened, whilst there is a corresponding swelling on the opposite side. It may readily be conceived that this alteration in the shape of the chest, which in fact contracts its dimensions, in all these varieties of deformity, is accompanied or followed by all "the symptoms that attend on an interrupted, quickened, or disordered circulation," (p. 69;) and that children labouring under this disease cannot run or quicken their pace like other children of the same age, without stopping frequently to take breath. After rather a long, but interesting, discussion relative to the causes of this deformity, our author proceeds to the curative means, with which we are more immediately concerned, and we shall give them in his own words:

"The method which I have employed with regard to the local means in those cases, where the spine has been exempt from disease, has been that of placing the intercostal muscles, and those connected with the anterior part of the chest, on the stretch, by placing the patient in a standing position, with the back against a cylindrical piece of wood, and the arms extended backwards. By this means an extension of the pectoral muscles is produced, and they are thus brought into full action upon the ribs, as well as the muscles of the abdomen which are opponents to them. The position, as well as the condition of the muscles, may be imagined by that of a person in the act of attempting to throw a somerset backwards. While the patient is in this situation, he is desired to take deep inspirations. I direct manipulation, and afterwards percussion, to be employed for one or two hours during the day, gradually increasing them in force, according to the influence produced on the patient."

Suspending the body by the arms, and similar modes of exercise, are recommended as additional means of cure, and assiduity and perseverance in this plan is earnestly enforced; and it is uniformly found, he says, that, in proportion as the parts are restored to their natural form, not only the respiration, but the action of the digestive organs, become more regular. Ten cases conclude this chapter.

The next chapter, upon *Contractions of the Limbs,* with four cases annexed, and which occupies a space of eighteen pages of the work, we shall not dwell upon, because there is no striking novelty, either of doctrine or practice; but we cannot too earnestly recommend to our junior brethren the necessity of perseverance in the use of the means of cure here advocated. Indeed, those who have not paid much attention to this subject, and have never witnessed the slow, but constant, advances towards recovery which muscular exertion produces in these instances, can have no idea of the extent of deformity which it is capable of remedying, and the very great benefits that result from its steady employment. Four additional cases are annexed to this chapter, giving an account of the removal of chronic affections of the muscles, either from falls or from the effect of rheumatism. The first of these cases is the more remarkable as the party was fifty years of age, and the fingers had been bent upon the palm of the hand for twelve months. The means used, independently of those medicines necessary to restore the digestive functions, which were deranged, consisted in manipulation and percussion over the extensor muscles for an hour each day, and the application of a splint inside the arm, with a spring attached to its extremity, the force of which was gradually increased, and they were taken off when pain was occasioned by their use. Seven weeks' perseverance in this plan restored the use of the wrist and fingers. (p. 110.)

We consider the chapter on *Paralysis* as the least satisfactory in this volume; and, of the six cases detailed, only one had reached to that period of life in which the employment of friction, percussion, and other topical remedies, could not be expected to have produced much effect; and it is not said that the restoration of the motion of the arm was by any means perfect, though certainly more than could have been expected under the circumstances. The other cases are chiefly children, and one where the palsy was induced by taking large quantities of laudanum.

With regard to the opinion quoted from Dr. COOKE, that paralytic affections are of rare occurrence among the soldiery, we have great doubt as to the fact; but of this we are sure, that those who have been soldiers suffer from palsy in as great, or even perhaps a greater, proportion than other people; and it must be recollected that men generally cease to be soldiers at, or soon after they are forty years of age, a period of life at which paralytic affections are rare in every class of society.

As the symptoms and mode of treatment of palsy are foreign to the purposes of this work, and as no new views are developed by our author, we shall pass them over, and proceed to the sixth chapter, on *Chorea St. Viti.* This is very short. The author gives it as his opinion that the practice recommended by Dr. HAMILTON is entitled to the greatest share of confidence; but, "in consequence of the beneficial results which had ensued from the employment of the exercise of volition in restoring the connexion between the sensorial and muscular power in paralytic affections," he was induced to try its influence in the cure of chorea; and four cases in illustration are given. In the first of these, a child of eleven years, the solutio arsenici and purgatives were regularly administered, and the degree of benefit derived from the muscular exercise of the arm is not, of course, to be accurately appreciated. The second case is much more satisfactory, because the subject of it (a boy between ten and eleven years old,) had for a long time been under treatment of various kinds, including arsenic and purgatives, together with exercise; and, as it was uncertain to which of these remedies the benefit derived was to be ascribed, Mr. Ward, after giving the medicines a fair, but unavailing trial for two months, left them off, and relied entirely upon the muscular exercise of the leg and arm. The patient began by holding a weight of four and a half pounds, with the arm extended, as long as he was able, and was ordered to repeat this several times in the course of the day. He was directed also to stand on the right leg only, as long as he was able, and to do so frequently in the day. In one month the convulsive motions entirely ceased.—The third case is remarkable for the convulsive motions having been suspended for a fortnight during the appearance of the nettle-rash; a circumstance which may afford a hint, on some future occasion, of trying the effect of an artificial eruption for the removal of similar symptoms. It appears very evident that in this instance the disease was connected with some affection of the stomach and bowels. It suffices to say that, by the use of muscular exertion, this case was also brought to a successful termination.

The volume concludes with a chapter of miscellaneous observations, recommending exercise in rheumatism,—noticing the diminished frequency of the pulse under the influence of muscular exercise,—and also urging the employment of friction, shampooing, &c. in gout; and suggesting the probability of curing certain imperfections of speech by the exercise of the muscles of respiration.

We take our leave of Mr. Ward by strongly recommending his work to the attention of professional men; and we can say, for our own parts, that we have long entertained views similar to those of our author relative to the advantages to be derived from perseverance in the means of cure which he has so ably advocated.

---

### DIVISION II.

### FOREIGN.

ART. II.—*De l'Hypochondrie et du Suicide, &c. &c.* Par J. P. FALRET, Docteur en Médecine de la Faculté de Paris, &c.—pp. 512. Paris, 1822.

THE work of M. FALRET, placed at the head of this article, consists of two separate, although not unconnected, essays,—the first on Suicide, the second on Hypochondriasis. Of these, it is our intention to begin with the latter, because we are inclined to believe that hypochondriasis frequently ends in that state of mind which leads to self-destruction; and, consequently, that the subject may be most advantageously treated by reversing the order of the author.

Two physicians, in Paris, of great promise, MM. GEORGET and FALRET, have devoted themselves to the same class of investigation in which PINEL and ESQUIROL have acquired such well-earned celebrity. Georget, in his treatise "sur la Folie," first departed from the received opinions regarding the seat of hypochondriasis as connected with the name of the disease, and placed it among affections of the brain; a position which he has subsequently more fully illustrated in his "Physiologie du Cerveau." Falret reviewed the former of these works in the "Journal

Complementaire," and supported the opinions of the author by further arguments and ingenious illustration. But still, as the review must necessarily follow the work reviewed, we cannot but smile at the embarrassment of M. Le Roy, who, alluding to this fact, is at a loss to know which of the two is entitled to the priority; thus seeking for M. Falret a merit which he was too ingenuous to claim for himself. The question is not one of much importance, but a considerate regard for the dilemma of M. Le Roy induces us to refer him to the work of Flemyng, published in 1741, where the same opinion will be found as distinctly stated as it is by either of the French pathologists.

It is somewhat singular that both Georget and Falret should have chosen to select M. Louyer Villermay as their opponent; and, as they have both followed nearly the same plan, we would have given the preference to the former, had not the essay of M. Falret been more full and particular. M. Villermay's doctrine is as follows:*—"Let us say then, along with the modern physiologists, that, from daily observation, and the attentive examination of the phenomena of the disease, that we recognize the abdominal viscera, and particularly the stomach, affected in their nervous system or their vital properties, and, above all, their organic sensibility, as the primary seat of hypochondriasis." The position of MM. Georget and Falret, on the contrary, is that this disease has always its seat in the brain, and that the lesion of any other organ can very seldom be regarded as the cause.

Before entering into a particular consideration of the disease, the author gives a short sketch of the grounds on which his opinion is founded, independently of morbid anatomy. An hereditary predisposition, great susceptibility, whether natural or acquired, and painful affections of the mind, it is remarked, are the ordinary causes of hypochondrism; and that all these act upon the brain, and through it upon other organs, is too obvious to require illustration. Another class of exciting causes, very analogous, is to be found in the society of invalids, and the perusal of medical books by persons not accustomed to such studies: these can only act upon the stomach indirectly, and so far we agree with M. Falret in his illustrations. Not so with that which follows. The professions, he says, which, without occupying the mind, require no locomotion, cause no waste of nervous influence, and so favour its accumulation in the brain, frequently giving rise to hypochondriasis, and "showing sufficiently well the seat of the disease." (p. 374.) This does not appear to us by any means good logic, for, in point of fact, it is begging the question; and it would be quite as good reason-

* *Traité des Maladies Nerveuses*, tom. i. p. 327.

ing to say, a certain degree of circulation is necessary to digestion, and exercise is necessary to effect this circulation; the mechanic has not exercise, therefore he does not digest. We acknowledge, however, that the solitary nature of some trades may have a tendency to produce habitual melancholy, and thus affect the stomach indirectly through the mind, to a degree which might not take place from the simple want of exercise. With regard to the other physical causes, our author thinks too much importance has been assigned them. Suppression of the menses, for instance, or hemorrhoids, he regards, not as the cause, but the effect, of a diseased state of the brain. No reason, however, is assigned by M. Falret for this opinion, and we are much inclined to receive it with distrust; for, however it may be in hypochondriasis, we are sure that the power of such suppressions to produce mischief in the head must require no proof —especially to any practitioner who has seen much of the diseases of women. Nothing is more frequent than to find violent head-ach brought on by sudden suppression of the catamenia, which becomes as suddenly relieved on their return.

These, however, are only to be regarded as introductory remarks, before the author proceeds to the more serious business of the essay; the general arrangement of which is as follows:— 1. He examines the causes with great attention, and endeavours to determine their nature and mode of action. 2. He relates the symptoms of hypochondriasis, in the order of their occurrence; such, at least, as he has observed. The progress and complications of the disease are also exposed. 3. That this arrangement may not appear arbitrary, he submits to analysis the facts in opposition to his own opinion, and endeavours to show that their natural explanation is favourable to him. 4. He details the result of morbid examinations; but, in place of establishing a general discussion on the seat of hypochondriasis, he contents himself with deducing the consequences which they present, according to the method employed with regard to the causes and symptoms. 5. He mentions his views of the distinctions admitted between hypochondriasis and some other diseases. 6. He details the means which appear best calculated to fulfil the indications taken from the state of the brain; the attention which the sympathies require; and, lastly, the treatment requisite to guard against an attack of hypochondriasis, or prevent its recurrence.

We now proceed to these heads in the order of their succession; and our readers are requested to keep in mind that, as the work of M. Louyer-Villermay contains the opinions most generally received, so the arguments of our author are especially pointed against it. Where this appears likely to cause ambiguity, we shall give the views of his opponent taken from his own words.

*Causes.*—The first and most striking of these is natural constitutional tendency to the disease; a doctrine not peculiar to M. Falret, but admitted by most writers on this subject, as Hoffman, Willis, Raulin, Laurent, Villermay, &c. Our author, however, goes further, and supposes the predisposing causes to be nearly the same as those of madness, and that an hereditary predisposition to mental alienation is very frequently a cause of hypochondriasis. From these circumstances the author endeavours to draw conclusions favourable to his leading position, that the brain is the seat of the disease; arguing that, as the symptoms of indigestion generally observed do not constitute hypochondriasis, unless combined with inquietude regarding the health, fickleness of purpose, and instability of character, so the hereditary predisposition must necessarily exist in the organ of the diseased functions,—viz. in the encephalon.

Persons possessing a nervous temperament, and gifted with a lively imagination, have always been regarded as peculiarly obnoxious to this disease. Rousseau, and our own elegant Cowper, form good illustrations. It is naturally to be expected that pursuits leading to the cultivation of fancy, or the indulgence of feeling, must prove powerful auxiliaries to the developement of hypochondriasis; which, accordingly, we find prevalent among all artists, but particularly musicians,—witness Viotti, Sacchini, Mozart, and others. Another proof of the effect of music upon minds endowed with undue sensibility, is observed in the melancholy and passionate desire of revisiting their native country produced among the Swiss soldiers on hearing the *Ranz des Vaches.* The long-continued cultivation of any study will frequently give rise to this complaint, although it is well ascertained that the mathematical sciences have less tendency to do so than others; the reason of which seems obviously to be, that they exercise principally the attention and judgment,—very sober qualities, when compared with the flights of unbridled imagination: indeed, the distinction is very well expressed by the name of "exact sciences," given to the former. These observations, though we believe them to be correct, are far from new, as we find Aristotle asking, "Cur homines qui ingenio claruerunt et in stadiis philosophiæ vel in republica administranda vel in carmine fingendo vel in artibus exercendis melancholicos omnes fuisse vedimus?"*

Women are said to be less liable to the disease than men; which may be accounted for, either by the fact of their wisely

* Aristot. *Prob.* sect. xxx.

avoiding to fatigue their minds by very profound meditations, or by supposing the same causes to produce a different series of phenomena, constituting hysteria,—a complaint, however, so analogous to the one of which we treat, that many have regarded them as the masculine and feminine of the same thing.

It would appear that climate has not much influence on hypochondriasis, which, however, bears a distinct relation to the progress of civilization, occurring particularly in those countries subject to great political events; a circumstance which accounts for the number of hypochondriacs observed by Zacchias in 1671, during the eventful reign of Louis the Fourteenth. The same effect is likewise said to have been produced in Spain and Germany, by the late invasions of the French. In this country, we have not had a similar opportunity of judging.

Sedentary habits are supposed to produce the disease, by preventing an equal distribution of nervous influence, and favouring its concentration in the principal source of sensibility. It is upon this principle, and not from their compressing the abdominal viscera, that M. Falret explains the occurrence of hypochondriasis among individuals following sedentary occupations. Were it not so, he thinks that the style of dress adopted by the female votaries of modern fashion would necessarily give rise to hypochondriasis. Now, without questioning the accuracy of M. Falret with regard to the degree of compression to which the "bas ventres" of his countrywomen are subjected, we must say that, in this country, the sufferings of the male sex in the cause of fashion must be quite as exquisite, —at least, if the apparent tightness of their stays be admitted as a sufficient criterion. We think our author refines a little too much on this point: if pressure be applied to the abdomen, so as to prevent a free distribution of nervous influence, must it not also prevent a free distribution of blood, and derange the functions of the viscera in various other ways?

The last, but not least frequent, of the predisposing causes is excess in venereal indulgences, particularly self-pollution.

According to M. Falret, the most powerful, or "almost the only causes, capable of giving rise to hypochondriasis, are intellectual or moral,—that is to say, cerebral. The exercise of the mental faculties, when it is not restrained within proper bounds, is a very powerful cause. This is so true, that it is infinitely rare to meet with one habitually given to study who has not experienced this affection, in a greater or less degree." (p. 387-8.) Besides the mere tendency produced by study in general, it is of every day's experience that particular trains of thought are more liable than others to excite the disease: such, for instance, is that caused by unprofessional persons reading medical books; and M. Villermay carries this so far as to lay down the habitual perusal of BUCHAN's Domestic Medicine *(lecture habituelle de Buchan,)* among the exciting causes. Rousseau describes, in natural and forcible language, the effect of such injudicious studies upon his singularly-constituted mind. He says, "Having read a little on physiology, I set about studying anatomy; and, passing in review the number and varied actions of the parts which composed my frame, I expected twenty times a-day to feel them going wrong. Far from being astonished to find myself dying, my astonishment was that I could live. I did not read the description of any disease, which I did not imagine myself to be affected with; and I am sure that, if I had not been ill, I must have become so from this fatal study. Finding in every complaint the symptoms of my own, I believed I had got them all, and thereby added another much more intolerable—the fantasie of curing myself: a thing difficult to avoid when one reads medical books. By means of plodding, reflecting, and comparing, I came to the conclusion that the root of my complaint was a polypus of the heart."*

The heart appears to be the part usually fixed upon by young medical hypochondriacs as the seat of disease, probably from the intimate sympathy this organ shows with all our mental feelings. We remember the late eminent Professor of Physic in Edinburgh used to mention in his lectures, that he was every season consulted very gravely by an immense number of young men on the state of their hearts; and M. Falret informs us, that when Corvisart drew the attention of the students at the Ecole de Médecine to the organic lesions of this organ, it brought on an epidemy of imaginary aneurisms.

The author proceeds to remark, that the passions hold the second rank in the production of hypochondriasis; and that this disease is particularly excited by painful affections of the mind,—as chagrin, fear, or ennui. "It is to this last cause, principally, that the hypochondriasis is to be referred which we so frequently remark in men of business who abandon their affairs, and, generally speaking, in those individuals who pass from an active life to one of idleness." It might have been necessary in this place, our author informs us, to prove that the passions have not their seat in the belly, "as is still in general supposed:" but we are convinced that, in this country at least, such a disquisition would have been regarded as altogether superfluous. Should there be any, however, who think arguments are necessary to prove this position, we refer them to the introduction to M. Falret's observations on mental derangement.

[The extent of the *Half-yearly Retrospect*, and of the interesting *Biography of* Dr. MARCET, (which we did not receive till the types had been set thus far,) oblige us to divide this article rather abruptly.]

* *Œuvres de* J. J. ROUSSEAU.

